# Modulation of gastrointestinal bacterial in chronic atrophic gastritis model rats by Chinese and west medicine intervention

**DOI:** 10.1186/s12934-021-01525-2

**Published:** 2021-02-02

**Authors:** Minghan Huang, Sihan Li, Youcheng He, Cuili Lin, Yueming Sun, Mingzhu Li, Rong Zheng, Ruoying Xu, Ping Lin, Xiao Ke

**Affiliations:** 1 Department of Gastroenterology, The Second People’s Hospital affiliated to Fujian University of Traditional Chinese Medicine, Fuzhou, 350003 China; 2 School of Basic Medical Sciences, Guangzhou University of Chinese Medicine, Guangzhou, 510006 China

**Keywords:** Chronic atrophic gastritis, Chinese medicine, Antibiotic, Dys-bacteriosis, Micro-ecology

## Abstract

Chronic atrophic gastritis (CAG) is well-known related with multiple pathogenic factors and normally therapies comprised by western or Chinese medicines. The present study was designed to identify the bacterial community characterized by 16S rRNA amplicon sequencing and determine the modulate affection of bacterial composition response western and Chinese medicine Qinghuayin (QHY) as well as antibiotic on model rats. The result shown the overall structure alteration of bacterial appeared under medicine intervened, antibiotic caused a marked depletion in bacterial diversity and richness. The enrichments of *Firmicutes* (85.1–90.7%) in antibiotic-free converts into *Bacteroidetes* (30.7–34.6%) in antibiotic-added model rat were demonstrated. *Firmicutes* as the most dominant phylum in antibiotic-free treatments and significantly decreased till 21.9–68.5% in antibiotic-added treatments. Especially QHY-treated rats showed highest RA of *Firmicutes* (90.7%) and the amelioration of CAG using QHY attributed by beneficial bacterial enrichment, especially *Ruminococcus*, *Lactobacillus* and *Bifidobacterium*. In addition, alpha and beta diversity analysis also demonstrated the clear dispersion and aggregation that revealed the alteration and steady of bacterial community structures. In summary, QHY has potential application value in the treatment of CAG, which attributed to close relation with the modulatory of internal bacterial communities.

## Introduction

Atrophic gastritis (AG) is chronic infection, and its primary symptoms are atrophy or/and gastric organs intestinal metaplasia. As soon as, the oxyntic mucosa is included, atrophy primes to the dearth of individually gastric acid and natural calculate generation in addition to iron or co-balamin malabsorption and in the long run anaemia. AG is a complex condition which will emerge from long-standing *Helicobacter pylori* (Hp) contamination or within the setting of immune system gastritis [13]. To date, no universally acknowledged criteria are accessible to characterize immune system gastritis and recognize such a clinical entity from incessant, *H. pylori*-driven, and multifocal atrophic gastritis (Rodríguez et al. 2017). Free epidemiological examination has affirmed that disease with *Hp* is the foremost imperative obtained aetio-logical mediator for gastric cancer, a worldwide driving basis of cancer associated deaths. The chronic inflammation initiated by *Hp* actuates a few histo-pathological variations within the gastric epithelium and keeps up a steady generation of a cascade of cytokines which in order to draw in resistant cells that create oxidative activists with the probable to harm host genetic materials. The instrument utilized by *Hp* in advancing the rise of pre-neoplastic gastric injuries (decay and intestinal metaplasia) is transcendently chronic inflammation [25].

The conformation of the gut microbiota is associated to illness, but the fact of distinct species is required to decode their biotic characters. The micro-biota investigations from the stomach are rare and observed that the human stomach was inhabited via a multipart microbiota counting basically *Firmicutes, Proteobacteria, Actinobacteria* and *Fusobacterium* phyla and appeared clear differences with micro-biota depicted within the mouth and esophagus [2]. Traditional Chinese medicine (TCM) has been connected with the prevention and treatment of stomach-related system issues in China for thousands of a long time [34]. Chronic atrophic gastritis (CAG) is a kind of the foremost common digestive system maladies in clinical practice, with assessed 50% of the world populace having the *Hp* contamination. These were required for compelling the medicate for chronic atrophic gastritis (CAG) with around 20% repeat rate [30]. Qinghuayin (QHY) is an important Chinese formulation, which is extensively utilized in the dealing of chronic atrophic gastritis (CAG) depend on the TCM clinical models of defrayal heat and determining dampness. QHY is self-possessed of herbs, and including *Baibiandou, Fuling, Yiyiren, Yinchen, Peilan, Baidoukou, Huanglian, Houpu,* and *Chishao* [12, 14]. A little Chinese investigation has described great effectiveness/protection of QHY in chronic atrophic gastritis (CAG) dealing. Li et al. [14] has confirmed the effectiveness of QHY as a TCM in a clinical rat trial model. The clinical trials indicated that TCM can neutralize gastric acid, decrease gastric acidity, pepsin action, unrestricted mucus, gastrin, TNF-α and IL-2 and then decrease inflammation in clinical trial of rats [40]. Furthermore, QHY has shown a good clinical efficacy in the clinical trial of Chen et al. [5]. TCM has not only prevent and treat infection and its recurrent attacks, but also has excellent clinical efficacy in improving body’s symptoms after infection. QHY is used as an adjuvant treatment against CAG, the process underlying QHY still necessitate additional investigation. In other cases, the fundamental components stay in unclear [14]. On the other hand, extraordinary advance has been made in explaining the pathogenesis of CAG, most western solutions, counting *Helicobacter pylori* (Hp) eradication, corrosive concealment, and non-steroidal anti-inflammatory drugs, as well as a stay un-satisfied. Due to chronicity and repeat of this illness, numerous sufferers have put their concentrations on elective medications such as conventional Chinese medicine [7].

The human gastro intestinal micro-biota could be a multifaceted micro system, comprising roughly 1014 organisms from over 1,000 microbes, of which the phyla *Bacteroidetes*, *Firmicutes* and *Actinobacteria* account for over 90% of the species. In expansion, the hereditary variables, dietary propensities, and different natural components of the host initiate the differences and the specificity of the intestinal microbes, whereas intestinal flora would perform vital parts in applying host physiological capacities, for example, digestion system, vitality ingestion, and safe control [38]. The intestine microbes are metabolized the TCM or co-metabolize TMC by the host,the produced metabolites have been shifting notches of bioavailability, bio-activity, and harmfulness. The configuration of the intestine micro-biota for homeostasis recuperation can moreover, be balanced usefully by TCM mechanisms. In this way, TCMs could enhance the dysfunction of the intestine micro-biota in conjunction with significant neurotic environments [17].

Within the earlier period, the analyst was endeavored to uncover the helpful system of Chinese drugs by recognizing bio-compounds in Chinese medicines utilizing cutting-edge science and innovation. In spite of much exertion, advance has been restricted. The standard worldview is highlighted the medicate revelation from Chinese drugs. These are included the herbal taking out, pre-fractionation, high-throughput study directed via disease-related targets (e.g., proteins, or RNA), testing models, and human trials. Utilizing such strategy, a few bio-compounds from Chinese medicine are undoubtedly established into present day drugs. Then, the trial rates could be exceptionally low. Many chemicals from Chinese medicines are demonstrated to be lacking or with small bio-action and bioavailability, and hence to avoid from moreover, dynamic substance of Chinese drugs or modern drug runners [43]. Therefore, the present study was design to identify the bacterial community characterized by 16S rRNA amplicon sequencing; and to determine the modulate affection of bacterial composition response western and Chinese medicine Qing huayin (QHY) as well as antibiotic on model rats.

## Materials and methods

### Preparation of test animals and medicinal

Fifty-nine specific pathogen free (SPF) male Wistar rats with a body mass of (110 ± 10 g) were purchased from Shanghai Laboratory Animal Co., Ltd., and the animal permit license number was SCXK (Shanghai) 2017–0005. They were raised in SPF animal Experiment Center of Fujian University of Traditional Chinese Medicine in separate cages with suitable ambient temperature (23 ± 2 °C) and air humidity (55 ± 5%).

The traditional Chinese medicine “Qinghuayin” was prepared by the Pharmacy Department of the Second People's Hospital affiliated to Fujian University of Traditional Chinese Medicine. Medicine composition was composed by atractylodes lancea (60 g), rhizoma coptidis (30 g), magnolia officinalis (60 g), artemisia capillaris (100 g), amomum cardamomum (30 g), paeonia obovata maxim (100 g), agastache rugosa (90 g), and coix lacryma-jobi (200 g). The decoction process including boiled for three times (1.5, 1 and 0.5 h respectively) after add eight times amount of water to soak for 0.5 h. Then combine the decoction and filtrated, the filtrate was decompressed and concentrated to 500 ml at 60–70 ℃. Finally, the concentration of the solution was 1.34 g/mL containing crude medicine, sterilized and packed then stored in refrigerator at 4 °C. The west medicine of ranitidine hydrochloride capsules (0.15 g/tablet) were produced by Sanofi (Hangzhou) Pharmaceutical Co., Ltd. (approval number: National Medicine Standard H33021741). The vitaco-enzyme tablets (0.2 g/tablet, approval number was National Medicine Standard H45021183) and experimental reagent of 1-methyl-3-nitro-1-nitrosoguanidine (number: OR301388) were produced by Beihai Sunshine Pharmaceutical and Bailingwei Technology Co., Ltd. The antibiotics ingredients including clindamycin hydrochloride, metronidazole, van-comycin hydrochloride, neomycin tri-sulfate hydrate and penicillin.

### Grouping and model construction

Specific pathogen free (SPF) Wistar rats were divided into 2 groups randomly, one group was composed by 8 rats and fed with SPF standard diet as control. For modeling group, 51 rats were feed in the normal environment by drink freely with 120 ug/ml 1-methyl-3-nitro-1-nitrosoguanidine solution, and gavage with 0.03 g/kg ranitidine daily and 45% ethanol twice a week on an empty stomach. At the same time, given feeding with starvation and satiety (fasting once a week, 24 h for one time) for 24 consecutive weeks. After the above model building intervention, the modeled rats gradually appeared unkempt and loose fur with yellow and dull, squinted eyes, dull activities, arched back, huddled and decreased appetite. At the end of 24 weeks, 3 rats were randomly sacrificed and stomach taken out then perform pathological examination to determine the success of CAG model.

### Medicine intervention, specimen preparation and collection

After the CAG model succeed construction, the modeled rats were divided into seven groups (each group has 8 rats) with antibiotic-free treatment of control, model (normal saline), western vitaco-enzyme and Chinese medicine, antibiotic-added treatment of model, western vitaco-enzyme and Chinese medicine. According to the equivalent dose conversion of clinical application, the traditional Chinese medicine group was gavage at the dose of the 5 ml/kg·d^−1^ QHY, and the solution was diluted with normal saline to 5 ml each time according to the weight of the rats. The western medicine group were gavage by suspension of vitaco-enzyme with diluted 0.2 g/kg and 5 ml each time. In the antibiotic treatment, added antibiotic while Chinese and western medicines were same as the above description. The control and model group were given 5 ml of normal saline each time by gavage. The medicine intervention was constant 30 days.

After the corresponding medicine intervention, the rats were fasted for 12 h and placed in transparent glass cover by a suitable size, then cotton ball soaked with ether was put into the glass cover to observe the rats breathing rhythm and movement situation. After successful anesthesia, the abdominal cavity was opened along the midline of the rat abdomen, and then separate the gastric and flush by normal saline, thereafter kept in frozen cryopreservation tube and quickly freeze in liquid nitrogen, finally stored in – 80 ℃ for analysis.

### Statistical analysis

Data shown are the means ± Standard deviation. T-test (prism 6.0) was used to analyze the data differences between the two groups. One way ANOVA (prism 6.0) was used to analyze the data difference and the significant (*p* < 0.05) during more than two treatments. The 16S rRNA microbial amplicon sequencing analysis was consistent with Zhang et al. [37] and performed by Allwegene Technologies Co., Ltd, China.

## Results and discussion

### Overall structural profile from phylum to genus in microbiota composition

**Fig. 1:**
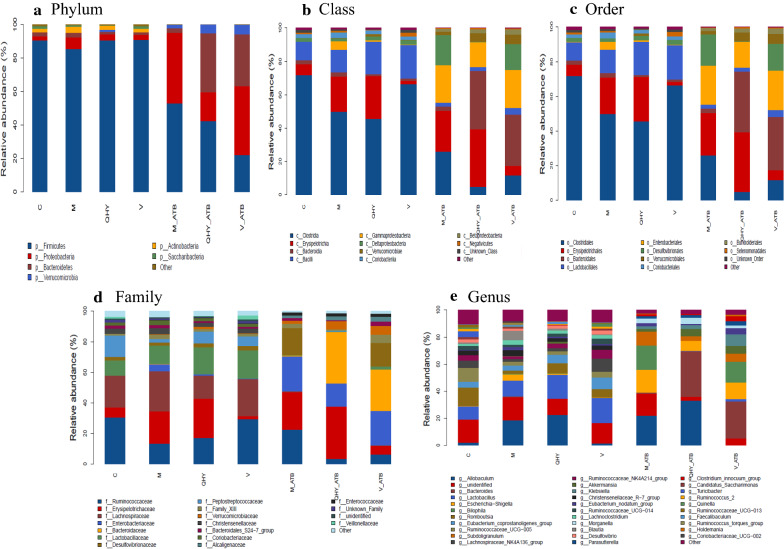
Histogram of relative abundance during phylum (**a**), class (**b**), order (**c**), family (**d**), and genus (**e**). The plotted by the "Relative Abundance" on the Y-axis and "Samples Name" on the X-axis. "Others" represents a total relative abundance of the top 10 phylum, class, order, family, genus and species

In order to identify the profile of specific group among the gastrointestinal microbial community in case of chronic atrophic gastritis (CAG) rats after medicine and antibiotic intervention. The relative abundance of the top taxa bacterial were identified from phylum to genes among all treatments (Fig. 1). At the phylum level, *Firmicutes* was most dominant position and accounting for 85.1%-90.5% in antibiotic-free treatments. Relatively higher relative abundance (RA) of *Firmicutes* were detected in medicine intervened group and QHY has richest abundance compare with control. The RA of *Proteobacteria* and *Actinobacteria* were obviously increased in M while similarly in other treatments. The RA of *Bacteroidetes* showed a certain level of reduction in medicine group compare with control (2.7–1.2%). While, distinct microbial distribution was descripted for antibiotic-added treatments, the RA of *Firmicutes* was observed significantly decreased till 21.9%-58.5%. The RA of *Proteobacteria* showed a clearly increased with richest RA presented in M_ATB and V_ATB. Considerably enhance of *Bacteroidetes* richness was illustrated in QHY_ATB (37.7%). The phylum of *Firmicutes* and *Bacteroidetes* were two major phyla in rats and humans and *Actinomycetes* was generally regarded as beneficial bacteria (e.g. *Bifidobacterium*) [29, 35]. The phylum of *Bacteroides* including potential pathogen that could be disturbed the immune function, and reduced richness in QHY treated rats revealed the inhabitation of potential pathogen of Chinese medicine.

At the class level, *Clostridia* was predominant in C, V, M, and QHY (45.6–71.7%), followed by *Erysipelotrichia* and richness in M and QHY. The RA of *Bacilli* was slightly increased in QHY (19.6%) than control (10.9%). On the contrary, *Erysipelotrichia* and *Bacteroidia* were dominant in antibiotic treatments, and the proportion of *Clostridia* significantly reduced (25.7–4.8%) and richest in M_ATB. The RA of *Erysipelotrichia* and *Bacteroidia* were sharply increased and richest in QHY_ATB. While the RA of *Bacilli* was decreased and similar abundance of *Gammaproteobacteria* and *Deltaproteobacteria* were increased as compare with non-antibiotic treatments. At the order level, *Clostridiales* was dominant in antibiotic-free treatments and QHY show lowest richness, followed by *Lactobacillales* was relatively increased in medicine added treatments, *Erysipelotrichales* was richer in QHY. In contrast, antibiotic-added treatments were dominated by *Erysipelotrichales* and *Bacteroidales* that richest in QHY_ATB while *Clostridiales* was dramatically reduced and the richness of *Enterbacteriales* and *Desulfovibrionales* were significantly increased. At the family level, *Ruminococcaceae* (29.9–35.5%), *Lachnospiraceae* (20.8–26.1%) and *Lactobaciillaceae* (18.5–17.6%) were dominant in antibiotic-free treatments. The proportion of *Enterobacteriaceae* and *Bacteroidacea* were increased and *Lactobacteriaceae* was decreased (< 2%) while the RA of *Erysipelotrichaceae* was clearly increased in antibiotic-added treatments and richest in QHY_ATB. When come to genus level, *unidentified* and *Lactobacillus* (11.7–18.5%) were dominant in antibiotic-free treatments. While antibiotic-added treatments were dominated by *Allobaculum* and *Bacteroides*, especially richness in QHY_ATB (55.7%). The genus of *Faecalibacterium* was known as functionally bacterial derived from salicylic acids and butyrate as well as anti-inflammatory molecule producing, *Faecalibacterium* and *Roseburia* were involved butyrate generating [21]. *Lactobacilli* was considered and well-known as probiotic and the RA were significantly decreased in antibiotic feed model rats that would be impair intestinal metabolism, similar with Jaan et al. [11] and Iino et al. [10].

**Fig. 2:**
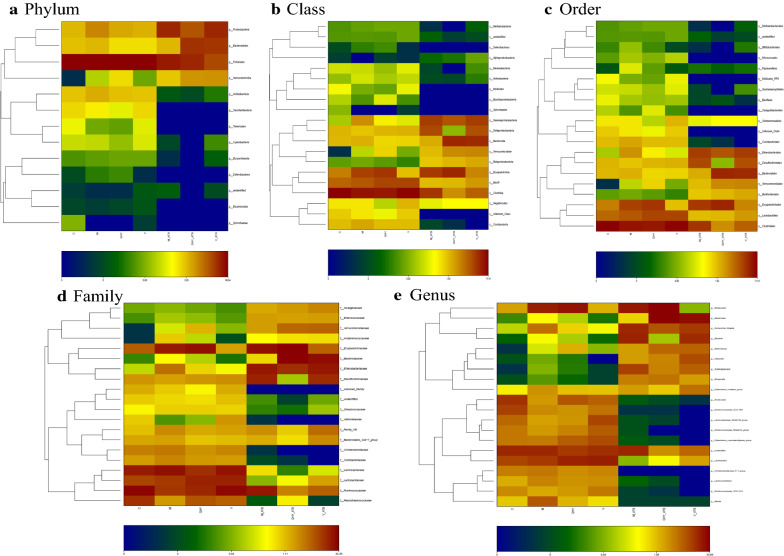
Heat-map based on taxonomy level: phylum (**a**), class (**b**), order (**c**), family (**d**), and genus (**e**). Different color means the different relative abundance of the genus in the all ten treatments

Consistently, Fig. 2 shows the distribution alteration of dominant bacterial in each treatment, there is obvious aggregation effect in the transformation of different medicine intervention. In the case of antibiotic-added treatments, the major proportion of bacterial phylum from *Firmicutes* convert into *Bacteroidetes* and *Proteobacteria*. Major class from *Clostridia* and *Bacilli* convert into *Bacteroidia*, *Gammaproteobacteria* and *Erysipelotrichia*. For order, *Clostridiales* and *Lactobacillales* were convert into *Bacteroidales*, *Enterobacteriales* and *Erysipelotrichales*. While *Lachnospiraceae*, *Lactobacillaceae* and *Ruminococcaceae* convert into *Enterobacteriaceae*, *Bacteroidaceae* and *Erysipelotrichaceae* at family level. Superior genus from *Lactobacillus* and *unidentified* convert into *Bacteroides*, *Allobalulum* and *Escherichia-Shigella*. Therefore, the heat map of dominant bacterial was altered by medicine intervene and antibiotic-added treatment showed the significantly different distribution pattern of bacterial community.

Chinese and western medicine-treated rats showed a certain level difference in bacterial diversity while antibiotic amendment caused a marked depletion in bacterial diversity and richness, which confirmed in previous report [9]. However, some specific bacterial were show increased patterns under reaction exposure on antibiotics in present study through all sequences detected. Antibiotic-added treatments shown lower RA of beneficial bacteria while higher RA of pathogenic bacterial like *Proteobacteria* that similar with Zhang et al. [41]. Treatment of QHY has highest population of beneficial bacterial of *Ruminococcus*, *Lactobacillus* and *Bacteroides*, while decreased abundance of *Desulfovibrio* and *Proteobacteria* that suggesting QHY might be maintain the micro-biota homeostasis.

### Classification display based comparison of phylum and genus levels

**Fig. 3:**
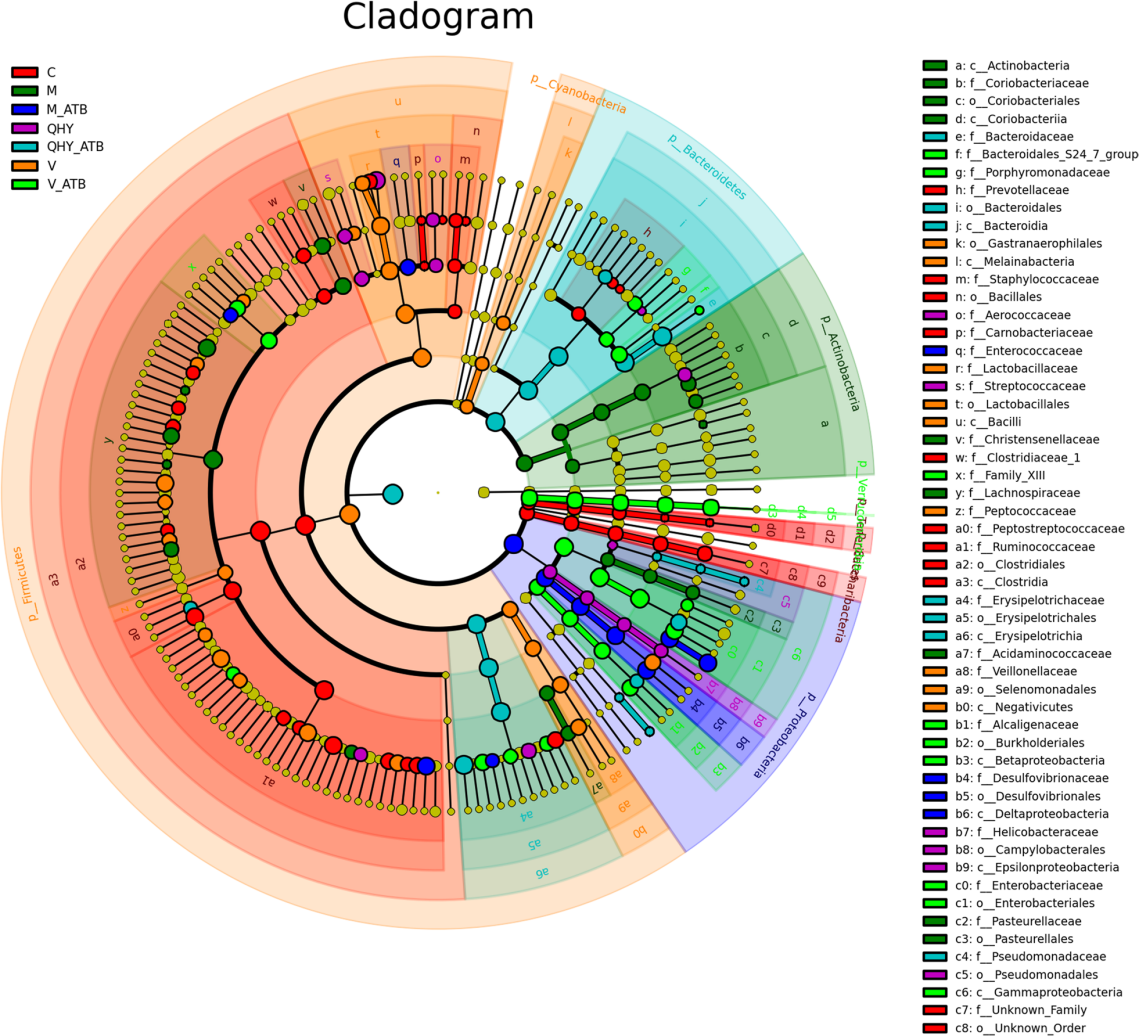
Linear discriminant analysis effect size cladogram. The color of the branch and the fan shape indicates its corresponding phylum and abundance, genes layout on different classification levels

The abundance and evolutionary relationship of genus are visually displayed in phylogeny and cladogram (Fig. 3 and Additional file 1: Fig. S1). The bacterial mainly distributed in *Firmicutes*, *Bacteroidetes* and *Proteobacteria* that consist with Aggeletopoulou et al. [1], while the composition of bacterial among distinct treatments presented a remarkably variations. To be specific, the dominant phylum of *Firmicutes* was richness with *Ruminococcaceae*, *Peptostreptococcaceae*, *Lachnospiraceae*, *Clostridiaceae_1*, *Lactobacillaceae* and *Staphylococcaceae*. The *Bacteroidetes* was richen in *Bacteroidaceae* and *Prevotellaceae*. The *Proteobacteria* was mainly distributed by *Enterobacteriaceae*, *Desulfovibrionaceae*, *Helicobacteraceae*, *Alcaligenaceae* and *Campylobacterales*. The phylum of *Actinobacteria* was distributed by *Coriobacteriaceae*. The phylum of *Cyanobacteria* was occupied by *Veillonellaceae* and *Gastranaerophilales*. It can be observed that the distribution of the bacterial community from distinct treatments were varied through discriminant transformation effect. Among the superior micro-biome phylotypes modulated by Chinese medicine and antibiotic-added intervene in rats. In this study results, we observed several putative beneficial genera containing *Lactobacillus*, *Paraprevotella* and *Eubacterium*. A pronounced depletion of *Clostridiales* and recovery of *Bacteroidales* were detected in antibiotic-added treatments. The RA of *Enterobacteriales* and *Verrucomicrobiales* were boosted at antibiotic-added treatment. Rats with antibiotic has obviously higher abundance of *Bacteroides* and lower *Clostridium* that may palliation the opportunities for pathogenic infection [18, 36, 39].

The remarkably proportion of *Lactobacillus* in antibiotic-free treatments than antibiotic-added group that might be related with the protective effect of Chinese medicine. Since long time, Chinese medicine has been considered as an important therapy to ameliorate adverse antibiotic reaction comprised diarrhea and dysbiosis [11, 32]. The main way for Chinese medicine to promote the proliferation of gut microbial is to play key role of prebiotic-like and selectively stimulating the metabolism of symbiotic beneficial bacterial, such as *Lactobacillus*, *Bacteroides* and *Bifidobacterium*. Various member of *Lactobacillus* has beneficial or positive effect on colitis and gastroenteritis model test [4, 22, 26]. In addition to having beneficial effects on host physiology, some component of symbiotic bacterial could be secrete molecules to limit microbial metabolism and proliferation [24]. Turroni et al. [28] found the alteration of *Lactobacillales* and *Bifidobacteriales* might be related to micro-biota components owing to enable to suppress the metabolic and proliferation of micro-biota through decreasing the number or diversity of observed genus and regulate microbial community structure [24]. Present finding obviously illustrated the intestinal biotransformation especially for beneficial microorganisms of CAG was observably strengthened by Chinese medicine.

### Diversity analysis based on alpha and beta

**Fig. 4:**
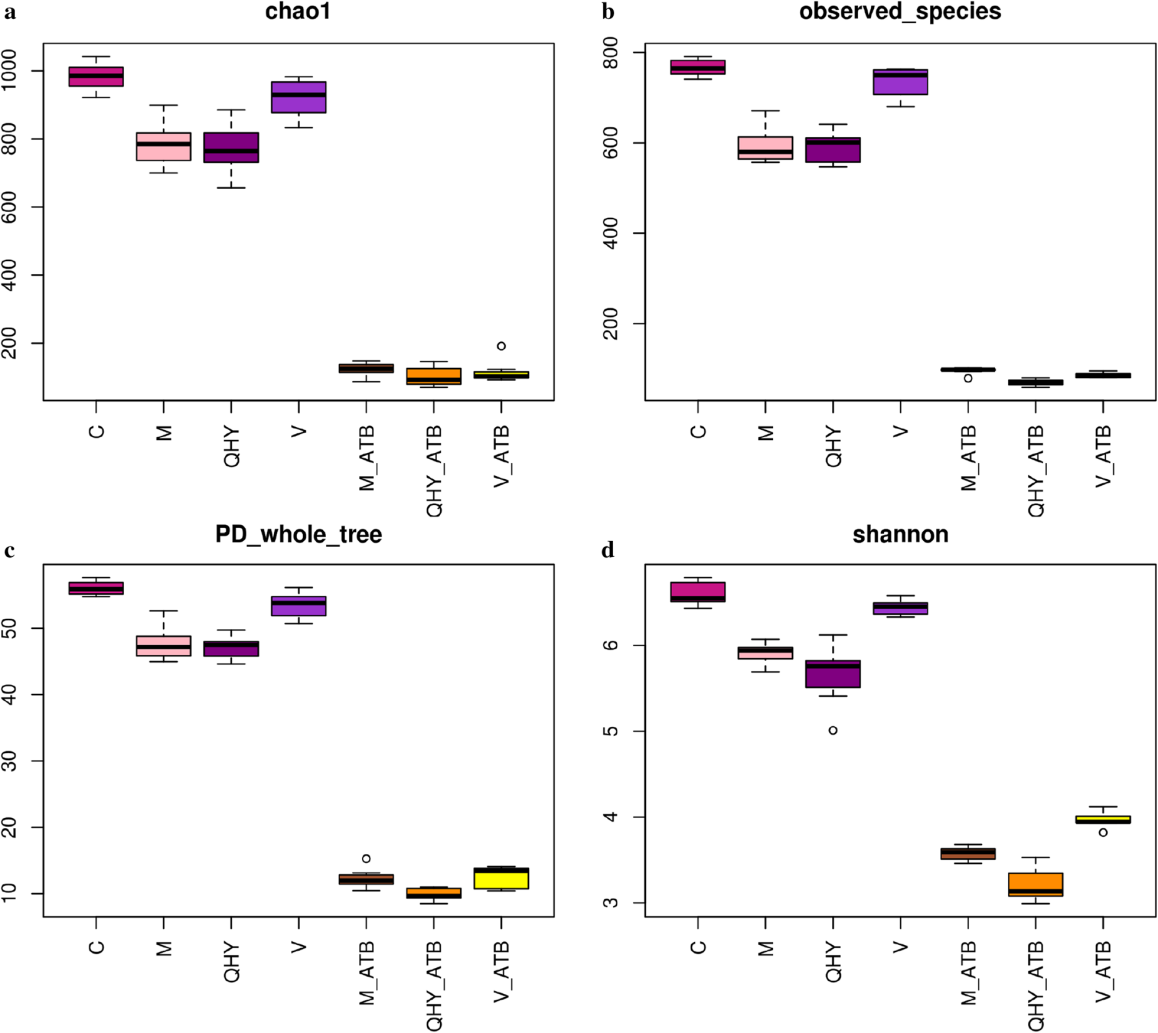
Alpha diversity within group differences based on chao1 (**a**), observed species (**b**), PD whole tree (**c**), shannon (**d**)

In order to comprehensively identify the changes of intestinal bacterial composition in CAG rat after interventions with normal saline, Chinese and western medicines as well as antibiotics, alpha and beta diversity analysis were performed to determine whether the community structure and steady state have changed (evaluated from the perspective of diversity and uniformity). The bacterial richness index based on the number of operational taxonomic units (OTUs) in the bacterial community was present in chao1 (Fig. 4a), 998, 765, 754, 965, 157, 122 and 146 were detected in C, M, QHY, V, M_ATB, QHY_ATB and V_ATB. The actually number of OTUs observed with the increase of sequencing depth was shown in observed species (Fig. 4b), 795, 587, 596, 773, 88, 72, and 81 were identified in the above treatments. The pedigree diversity based bacterial abundance and evolutionary distance were performed in PD whole tree (Fig. 4c). The result shown 58, 46, 45, 51, 13, 9, and 12 were discovered in C, M, QHY, V, M_ATB, QHY_ATB and V_ATB. As well as shannon demonstrated 7.5, 5.9, 5.6, 6.4, 3.5, 3.2, and 4 were present in above treatments (Fig. 4d). Therefore, bacterial diversity of antibiotic-added treatments was obviously decreased. The Venn figure also verified the consistent results (Additional file 1: Fig. S2) and indicated the metabolism conspicuously weakened by antibiotic intervention.

**Fig. 5:**
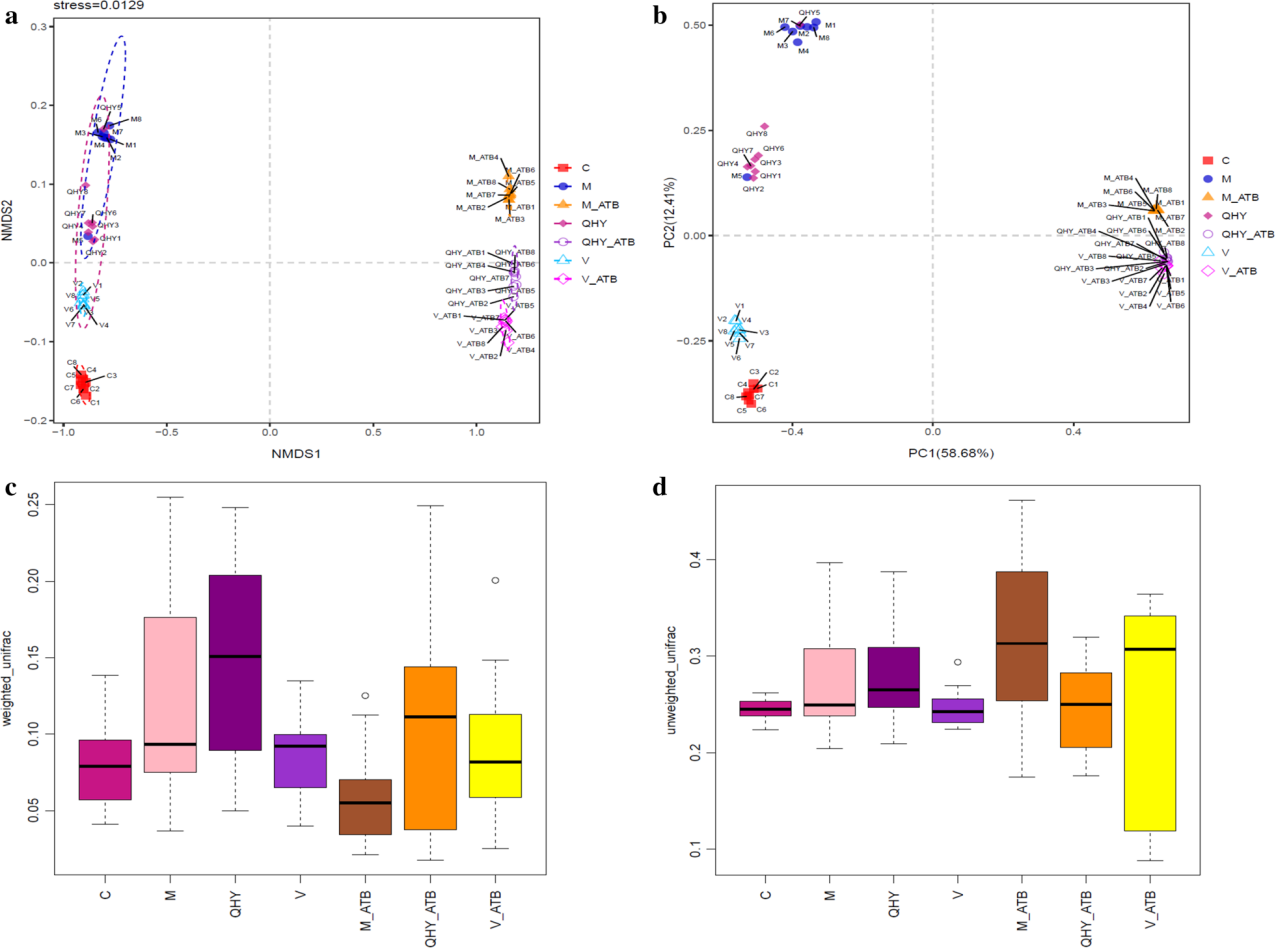
Beta diversity within group differences based on **a** non-metric multi-dimensional scaling analysis; **b** principal component analysis; **c** box plot based on diversity of weighted and **d** unweighted unifrac distance

The beta diversity aspect of non-metric multi-dimensional scaling (Fig. 5a) and principal component analysis (Fig. 5b) were carried out to compare the bacterial community distribution, further demonstrated an obviously separation among different treatments of overall bacterial structure in rat after medicine and antibiotics treated. The principal components were account for 58.68% and 12.41% of the total variation respectively. Treatments of C and V were clustered one group, M and QHY were gathered while M_ATB distributed individually one group, QHY_ATB gather together and have intersection with V_ATB. In addition, weighted (Fig. 5c) and un-weighted unifrac (Fig. 5d) also demonstrated the differences among all treatments. The degree of dispersion between the medicine and the antibiotic-added group were different significantly. The west and Chinese medicine group has an overlap and large dispersion with antibiotic group. The affection of antibiotics is mainly focus on reduce the diversity and richness of beneficial intestinal microorganisms, aggravate the intestinal microbial imbalance and increase the probability of the intestinal pathogen invasion and attachment, further caused more serious damage to the intestinal barrier function [3, 11]. The diversity of gastrointestinal microbial composition enables to resist the adverse altered in the environment and recover equilibrium after perturbation (resistance and resilience), which was positively facilitated by Chinese medicine.

### Correlation analysis among superior genus based on network

**Fig. 6:**
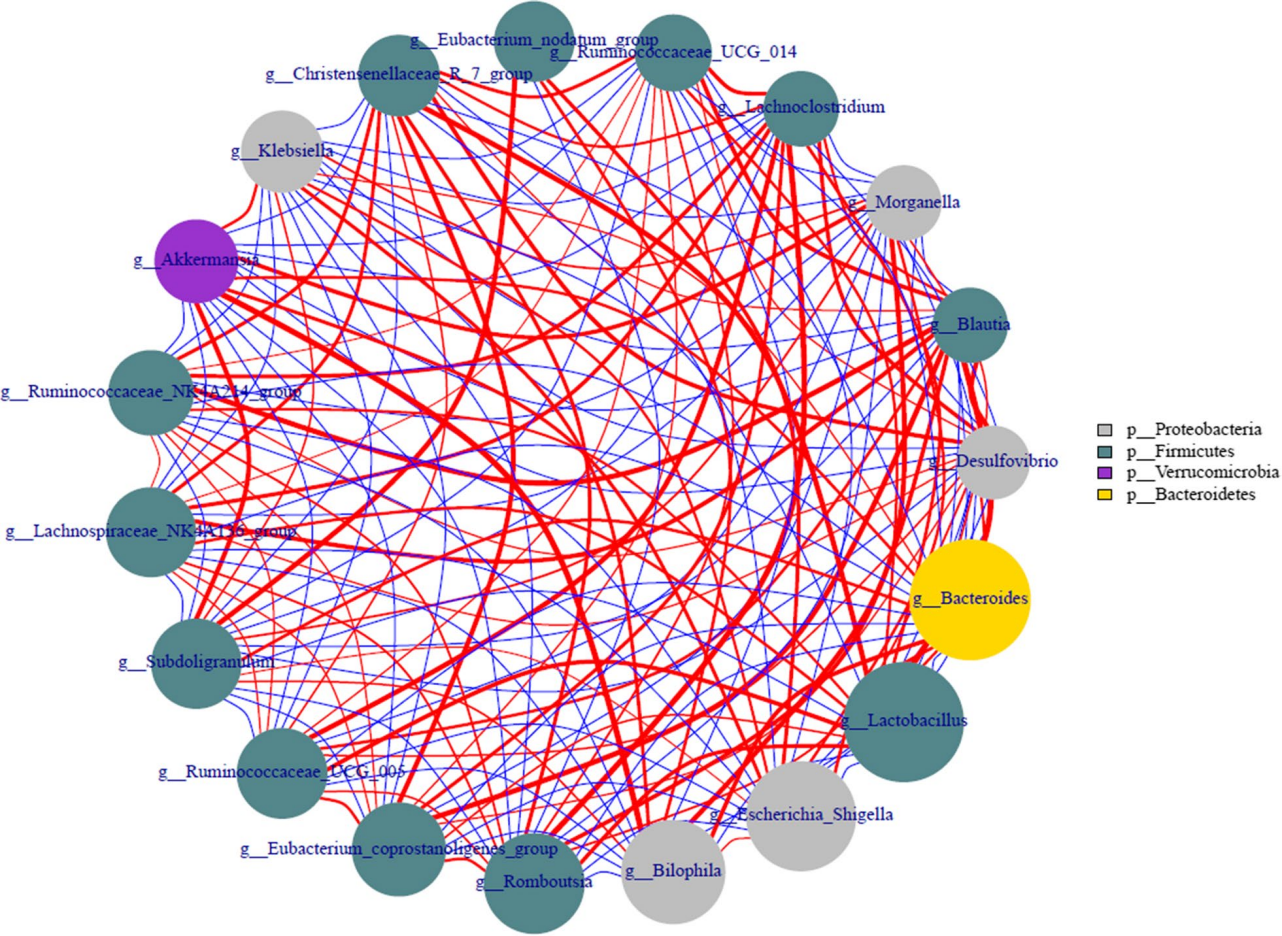
Correlations among richness genus based on network. Red line represents positive correlation and blue line represents negative correlation, the thickness of the line represents the strength of the correlation and the area of the gene circle represents the relative abundance. *C* control, *M* model with normal saline, *QHY* traditional Chinese medicine Qinghuayin, *V* western medicine vitacoenzyme, *V_ATB* western medicine vitacoenzyme and antibiotic added, *M_ATB *model with normal saline and antibiotic added, and *QHY_ATB* traditional Chinese medicine Qinghuayin and antibiotic added

The intestinal microecology plays essential role aspect of health and fight disease and ecological disorders accounting for great proportion in pathophysiology. Interestingly, microorganisms play an essential contribution in maintain the immune system homeostasis steady state and the niche-specific microbial network can be reflected the intestinal microenvironment [6, 11]. Coker et al. [6] observed the co-occurrence and co-exclusion interaction between GC enrichment and replacement bacteria were increasing with disease progression and external factor intervention. To be specific, present study was indicated that intricate correlations among richness genus, *Lactobacillus* was strongly positive with *Ruminococcaceae_UCG_005* and *Christensenellaceae_R7*, negatively with *Subdoligranulum*, *Klebsiella* and *Lachnoclostridium*. The bacterial of *Bacteroides* was positively with *Klebsiella*, *Akkermansia* and *Subdoligranulum*, negatively with *Eubacterium_coprostanoligenes* and *Lachnospiraceae_NK4A136*. The genus of *Bilophila* with *Subdoligranulum*, *Akkermansia* and *Eubacterium_nodatum* shown a strong negative correlation. In addition, genus of *Blautia* was positively correlated with *Ruminococcaceae_UCG_014*, *Ruminococcaceae_NK4A214, Lachnospiraceae_NK4A136* and *Eubacterium_coprostanoligenes*, negatively with *Klebsiella*, *Akkermansia* and *Subdoligranulum*. *Akkermansia* has positively related with *Bilophila*, *Subdoligranulum*, and *Morganella*, negatively related with *Ruminococcaceae* and *Lachnospiraceae* (Fig. 6).

In view of *Blautia* can be convert carbohydrates and proteins to acetic acid and further responsible for energy supply. While many researches demonstrated the RA of *Blautia* was increased in diseases condition such as diabetes, irritable bowel syndrome due to it can be activate inflammatory cytokines [33], which is same with present study and the RA was obviously increased in M-treated. The genus of *Akkermansia* as a mucus-degrading bacterial that exists in the mucus layer, which enhance the integrity of the intestinal barrier and regulate intestinal metabolism [8, 20]. The genus of *Clostridium* is the main bacteria producing butyric acid and further fermentation convert to short-chain fatty acids that play an important role in maintaining host health and disease prevention. It can provide the host colonic epithelial cells and promote the growth of intestinal epithelial cells, accelerate the repair of damaged intestinal mucosa, also physiologically regulate the gene expression of intestinal epithelial cells that effectively inhibit the occurrence of enteritis and colorectal cancer. The genus of *Enterococcus* can produce tyramine that was related to tyrosine metabolism and can enhance the adhesion of bacteria to the intestinal, further improving colonic mucosal adhesion [11]. The RA of *Desulfovibrio* and *Bilophila* were increased and indicated that the selective specific bacterial community correlated with inflammation has altered in case of antibiotic application [27].

A Chinese medicine intake has been shown to trigger gut dysbiosis, increase intestinal permeability and alter gut microbiota composition [23, 31]. The mechanism of Chinese medicine action on the weakened spleen and stomach CAG was mainly to protect the gastric mucosa and reduce inflammation, improve gastric mucosal secretion and regulate gastrointestinal motility, the curative effect is accurate and the safety is high [15], [16]). Zhou et al. [42] verified Chinese medicine ginseng stimulated the growth of crucial probiotics *Lactobacillus* and *Bacteroides*. The increased number of beneficial bacterial in the intestines *Lactobacillus* was great significance for inhibiting the proliferation of spoilage bacterial and enhancing the immunity as well as resistance of the body. Interestingly, Chinese medicine intervention restored the microbial community perturbed by CAG holistically that consistent with Zhou et al. [42] and the mechanisms involved can be intricate. After the intervention of antibiotics, the specific bacterial such as *Enterococcus* multiplies that is related to the survival requirement and colonization mechanism of the organism [19]. When long-term use of antibiotics might be caused gastrointestinal diseases and showing a state of intestinal micro-ecosystem disorders, owing to pathogens such as *Escherichia coli* will proliferate while probiotics like *Bifidobacterium*, *Lactobacillus*, and *Bacteroides* were reduced significantly. At the same time, the imbalance of microbial will in turn affect the absorption of nutrients, reduce the immunity, weaken the intestinal barrier function, and further aggravate the disease.

This study suggests that the total number of bacteria and *Lactobacillus* during the treatment of QHY have shown a growth trend, chronic atrophic gastritis disrupts the balance of the normal microbial community, while the application of QHY can recover new balance and more optimized by improving the diversity of the intestinal microbial. Therefore, this phenomenon possibly has great potential to contributed for the alleviation of clinical inflammation and intestinal microenvironmental homeostasis, which conductive to understanding and apply the effective and promising approach of microbial-specific targeted therapy while its metabolic pathway of action needs to be further studied.

## Conclusion

The gastrointestinal bacterial composition was obviously different of the CAG model rats under the medicine intervention. Antibiotic treated model rats were remarkably decreased the abundance and diversity of bacterial community, with the depletion of *Firmicutes* (85.1%-20.3%) and expanded the *Bacteroides* (37.7% in QHY_ATB). Chinese medicine QHY prominently ameliorated GCA induced gastrointestinal injury by promoting effect on the proliferation of probiotics *Lactobacillus* (11.7–18.5%), which effect on protection of the gastric mucosa barrier and beneficial regulation of the microbial, while its metabolic pathway of action needs further study.

## Supplementary Information

**Additional file1**: **Fig. S1**: Phylogenetic tree constructed by the representative sequence of the horizontal species; the color of the branch and the fan shape indicates its corresponding gate, circles from inside to outside stand for system composition tree, genes layout on different classification levels (represent by different color, and the area of sector means respective proportion), the stacked column chart outside the fan ring indicates the abundance distribution information of the genus in different treatments. **Fig. S2**: Venn figure during treatments. C(control), M(model with normal saline), QHY(traditional Chinese medicine Qinghuayin), V(western medicine vitacoenzyme), V_ATB(western medicine vitacoenzyme and antibiotic added), M_ATB(model with normal saline and antibiotic added), and QHY_ATB(traditional Chinese medicine Qinghuayin and antibiotic added) (DOC 2717 KB)

## References

[1] Aggeletopoulou I, Konstantakis C, Assimakopoulos SF, Triantos C (2019). The role of the gut microbiota in the treatment of inflammatory bowel diseases. Microb Pathogenesis.

[2] Aviles-Jimenez F, Vazquez-Jimenez F, Medrano-Guzman R, Mantilla A, Torres J (2014). Stomach microbiota composition varies between patients with non-atrophic gastritis and patients with intestinal type of gastric cancer. Sci Rep.

[3] Brugiroux S, Beutler M, Pfann C, Garzetti D, Ruscheweyh H, Ring D, Diehl M, Herp S, Lötscher Y, Hussain S, Bunk B, Pukall R, Huson D, Münch P, McHardy A, McCoy K, Macpherson A, Loy A, Clavel T, Berry D, Stecher B (2016). Genome-guided design of a defined mouse microbiota that confers colonization resistance against *Salmonella enterica* serovar Typhimurium. Nat Microbiol.

[4] Chang HY, Chen JH, Chang JH, Lin HC, Lin CY, Peng CC (2017). Multiple strains probiotics appear to be the most effective probiotics in the prevention of necrotizing enterocolitis and mortality: an updated meta-analysis. PLoS ONE.

[5] Chen T (2017). Clinical efficacy observation of combination of traditional Chinese medicine and western medicine in treatment of 92 cases of *HP* infected chronic atrophic gastritis. Biomed Res.

[6] Coker O, Dai Z, Nie Y, Zhao G, Yu J (2017). Mucosal microbiome dysbiosis in gastric carcinogenesis. Gut.

[7] Dai Y, Zhang Y, Li D, Ye J, Zeng L, Wang Q, Hu L, Lin Y (2017). The efficacy of Jianpi Yiqi therapy for chronic atrophic gastritis: a systematic review and meta-analysis. PLoS ONE.

[8] Everard A, Belzer C, Geurts L, Ouwerkerk JP, Druart C, Bindels LB, Guiot Y, Derrien M, Muccioli GG, Delzenne NM, De Vos WM, Cani PD (2013). Cross-talk between *Akkermansia muciniphila* and intestinal epithelium controls diet-induced obesity. Proc Natl Acad Sci USA.

[9] Fröhlich EE, Farzi A, Mayerhofer R, Reichmann F, Jačan A, Wagner B, Zinser E, Bordag N, Magnes C, Fröhlich E, Kashofer K, Gorkiewicz G, Holzer P (2016). Cognitive impairment by antibiotic- induced gut dysbiosis: analysis of gut microbiota–brain communication. Brain Behav Immun.

[10] Iino C, Shimoyama T, Chinda D, Arai T, Chiba D, Nakaji S, Fukuda S (2018). Infection of helicobacter pylori and atrophic gastritis influence *Lactobacillus* in gut microbiota in a Japanese population. Front Immunol.

[11] Jaan A, Kashofer K, Zenz G, Frhlich EE, Reichmann F, Hassan AM, Holzer P (2020). Synergistic and antagonistic interactions between antibiotics and synbiotics in modifying the murine fecal microbiome. Springer Open Choice.

[12] Ke X, Zhou F, Gao Y, Xie B, Hu G, Peng W, Chen J, Sferra T (2013). Qing Hua Chang Yin exerts therapeutic effects against ulcerative colitis through the inhibition of the TLR4/NF-κB pathway. Int J Mol Med.

[13] Lahner E, Carabotti M, Annibale B (2018). Treatment of Helicobacter pylori infection in atrophic gastritis. J Gastroenterol.

[14] Li S, Huang M, Chen Q, Li S, Wang X, Lin J, Zhong G, Lin P, Asakawa T (2018). Confirming the effects of qinghuayin against chronic atrophic gastritis and a preliminary observation of the involved inflammatory signaling pathways: an in vivo study. Evid Based Complement Alternat Med.

[15] Li S, Li J (2020). Treatment effects of Chinese medicine (Yi-Qi-Qing-Jie herbal compound) combined with immunosuppression therapies in IgA nephropathy patients with high-risk of end-stage renal disease (TCM-WINE): study protocol for a randomized controlled trial. Trials.

[16] Liu H, Zheng J, Lai H, Hu B, Zhu L, Leung E, Wei H (2020). Microbiome technology empowers the development of traditional Chinese medicine. Sci China Life Sci.

[17] Lu Y, Xie J, Peng C, Wang B, Wang K, Li L (2019). Enhancing clinical efficacy through the gut microbiota: a new field of traditional Chinese medicine. Engineering.

[18] Lv W, Liu C, Ye C, Sun J, Tan X, Zhang C, Qu Q, Shi D, Guo S (2017). Structural modulation of gut microbiota during alleviation of antibiotic-associated diarrhea with herbal formula. Int J Biol Macromol.

[19] Ferrer M, Santos VAPM, Ott SJ, Moya A (2014). Gut microbiota disturbance during antibiotic therapy. Gut Microbes.

[20] Ottman N, Reunanen J, Meijerink M, Pietilä TE, Kainulainen V, Klievink J, Huuskonen L, Aalvink S, Skurnik M, Boeren S, Satokari R, Mercenier A, Palva A, Smidt H, de Vos WM, Belzer C (2017). Pili-like proteins of Akkermansia muciniphila modulate host immune responses and gut barrier function. PLoS One.

[21] Ou Y, Chen S, Ren F, Zhang M, Ge S, Guo H, Zhang H, Zhao L (2019). *Lactobacillus casei* strain shirota alleviates constipation in adults by increasing the pipecolinic acid level in the gut. Front Microbiol.

[22] Rodríguez NA, Algieri F, Garrido M, Vezza T, Utrilla MP, Chueca N, Garcia F, Olivares M, Rodríguez C, Gálvez J (2017). Differential intestinal anti-inflammatory effects of *Lactobacillus fermentum* and *Lactobacillus salivarius* in DSS mouse colitis: impact on microRNAs expression and microbiota composition. Mol Nutr Food Res.

[23] Su Z, Lin Z, Chen P, Tung Y (2019). Delirium: Traditional Chinese medicine herbs and gut microbiota. Ann Adv Biomed Sci.

[24] Suez J, Zmora N, Zilberman-Schapira G, Mor U, Dori-Bachash M, Bashiardes S, Zur M, Regev LD, Ben Z, Federici S, Horn M, Cohen Y, Moor AE, Zeevi D, Korem T, Kotler E, Harmelin A, Itzkovitz S, Maharshak N, Shibolet O, Pevsner FM, Shapiro H, Sharon I, Halpern Z, Segal E, Elinav E (2018). Post-antibiotic gut mucosal microbiome reconstitution is impaired by probiotics and improved by autologous FMT. Cell.

[25] Sung JJY, Coker OO, Chu E, Szeto C, Luk S, Lau H, Yu J (2020). Gastric microbes associated with gastric inflammation, atrophy and intestinal metaplasia 1 year after Helicobacter pylori eradication. Gut.

[26] Szajewska H, Kołodziej M, Gieruszczak-Białek D, Skórka A, Ruszczyński M, Shamir R (2019). Systematic review with meta-analysis: *Lactobacillus rhamnosus* GG for treating acute gastroenteritis in children—a 2019 update. Aliment Pharmacol Ther.

[27] Tang W, Yao X, Xia F, Yang M, Chen Z, Zhou B, Liu Q (2018). Modulation of the gut microbiota in rats by hugan qingzhi tablets during the treatment of high-fat-diet-induced nonalcoholic fatty liver disease. Oxid med cell longev..

[28] Turroni F, Ventura M, Buttó LF, Duranti S, O’Toole PW, Motherway MO, van Sinderen D (2014). Molecular dialogue between the human gut microbiota and the host: a *Lactobacillus* and *Bifidobacterium* perspective. Cell Mol Life Sci.

[29] Wei X, Tao J, Xiao S, Jiang S, Shang E, Zhu Z (2018). Xiexin tang improves the symptom of type 2 diabetic rats by modulation of the gut microbiota. Sci Rep..

[30] Wei Y, Ma L, Yin S, An J, Wei Q, Yang J (2015). Huangqi Jianzhong Tang for treatment of chronic gastritis: a systematic review of randomized clinical trials. Evid Based Complement Alternat Med.

[31] Xiao S, Fei N, Pang X, Shen J, Wang L, Zhang B, Zhang M, Zhang X, Zhang C, Li M, Sun L, Xue Z, Wang J, Feng J, Yan F, Zhao N, Liu J, Long W, Zhao L (2014). A gut microbiota-targeted dietary intervention for amelioration of chronic inflammation underlying metabolic syndrome. FEMS Microbiol Ecol..

[32] Xu J, Chen H, Li S (2017). Understanding the molecular mechanisms of the interplay between herbal medicines and gut microbiota. Med Res Rev.

[33] Yan X, Feng B (2016). Microflora disturbance during progression of glucose intolerance and effect of sitagliptin: an animal study. J Diabetes Res..

[34] Yang L, Li J, Hu Z, Fan X, Pan H (2020). A systematic review of the mechanisms underlying treatment of gastric precancerous lesions by traditional Chinese medicine. Evid Based Complement Alternat Med..

[35] Zareef R, Younis N, Mahfouz R (2020). Inflammatory bowel disease: a key role for microbiota?. Meta Gene..

[36] Zhang J, Guo Z, Xue Z, Sun Z, Zhang M, Wang L, Wang G, Wang F, Xu J, Cao H, Xu H, Lv Q, Zhong Z, Chen Y, Qimuge S, Menghe B, Zheng Y, Zhao L, Chen W, Zhang H (2015). A phylo-functional core of gut microbiota in healthy young Chinese cohorts across lifestyles, geography and ethnicities. ISME J.

[37] Zhang M, Fan X, Fang B, Zhu C, Zhu J, Ren F (2015). Effects of *Lactobacillus salivarius* Ren on cancer prevention and intestinal microbiota in 1,2-dimethylhydrazine-induced rat model. J Microbiol.

[38] Zhang R, Gao X, Bai H, Ning K (2020). Traditional Chinese Medicine and gut microbiome: their respective and concert effects on healthcare. Front Pharmacol.

[39] Zhang SY, Zhao FJ, Sun GX, Su JQ, Yang XR, Li H, Zhu YG (2015). Diversity and abundance of arsenic biotransformation genes in paddy soils from southern China. Environ Sci Technol.

[40] Zhang X, Chen W, She B, Luo R, Shi N, Xue P, Yang X, Xia Q (2014). The efficacy and safety of Jian-Wei-Qu-Tong Pills for the treatment of chronic non-atrophic gastritis (spleen and stomach qi deficiency with damp-heat stasis syndrome): study protocol for a phase II, randomized controlled trial. Trials.

[41] Zhang X, Yuan Z, Qu C, Yu X, Huang T, Chen PV, Su Z, Dou Y, Wu J, Zeng H, Xie Y, Chen J (2018). Palmatine ameliorated murine colitis by suppressing tryptophan metabolism and regulating gut microbiota. Pharmacol Res.

[42] Zhou S, Xu J, Zhu H, Wu J, Xu D, Yan R, Xiu Y, Huan L, Su M, Zhuo W, Hu C, Hong S, Song L (2016). Gut microbiota-involved mechanisms in enhancing systemic exposure of ginsenosides by coexisting polysaccharides in ginseng decoction. Sci Rep.

[43] Zhou Z, Chen B, Chen S, Lin M, Chen Y, Jin S, Chen W, Zhang Y. Applications of network pharmacology in traditional chinese medicine research. *Evid.-Based Complementary Altern. Med.* 2020; 1–7. 10.1155/2020/1646905 PMC7042531 32148533

