# Supplementary material for: Modulation of gastrointestinal bacterial in chronic atrophic gastritis model rats by Chinese and west medicine intervention
Source: Microb Cell Fact. 2021 Feb 2;20:31. doi: 10.1186/s12934-021-01525-2 (PMC7852297; doi:10.1186/s12934-021-01525-2)
Supplement: Supplementary file 1 — Additional file1: Fig. S1: Phylogenetic tree constructed by the representative sequence of the horizontal species; the color of the branch and the fan shape indicates its corresponding gate, circles from inside to outside stand for system composition tree, genes layout on different classification levels (represent by different color, and the area of sector means respective proportion), the stacked column chart outside the fan ring indicates the abundance distribution information of the genus in different treatments. Fig. S2: Venn figure during treatments. C(control), M(model with normal saline), QHY(traditional Chinese medicine Qinghuayin), V(western medicine vitacoenzyme), V_ATB(western medicine vitacoenzyme and antibiotic added), M_ATB(model with normal saline and antibiotic added), and QHY_ATB(traditional Chinese medicine Qinghuayin and antibiotic added) (DOC 2717 KB) [file 12934_2021_1525_MOESM1_ESM.doc]

**SUPPLEMENTARY FILE**

**Modulation of gastrointestinal bacterial in chronic atrophic gastritis model rats by Chinese and west medicine intervention**

**Minghan Huang ^#1^, Sihan Li ^#2,*^, Youcheng He^#1^, Cuili Lin^1^, Yueming Sun^1^, Mingzhu Li^2^, Rong Zheng^1^, Ruoying Xu^1^, Ping Lin^1,*^, Xiao Ke^1,*^**

*^1^Department of Gastroenterology, the second people's Hospital affiliated to Fujian University of traditional Chinese Medicine, Fuzhou 353003, China*

*^2^School of Basic Medical Sciences, Guangzhou University of Chinese Medicine, Guangzhou 510006, China*

**^#^** equally contributing author

***Corresponding author:**

**Dr. Sihan Li,**

Email: 20182104116@stu.gzucm.edu.cn

**Prof. Ping Lin,**

Email: drfjlinping@163.com

**Prof.** **Xiao Ke,**

Email: drkxkx@163.com

**Figure captions:**

**Fig. S1.** Phylogenetic tree constructed by the representative sequence of the horizontal species; the color of the branch and the fan shape indicates its corresponding gate, circles from inside to outside stand for system composition tree, genes layout on different classification levels (represent by different color, and the area of sector means respective proportion), the stacked column chart outside the fan ring indicates the abundance distribution information of the genus in different treatments.

**Fig. S2.** Venn figure during treatments. C(control), M(model with normal saline), QHY(traditional Chinese medicine Qinghuayin), V(western medicine vitacoenzyme), V_ATB(western medicine vitacoenzyme and antibiotic added), M_ATB(model with normal saline and antibiotic added), and QHY_ATB(traditional Chinese medicine Qinghuayin and antibiotic added).

**Figure S1.**

**Figure S2.**
